# Recent Advances in MXene-Based Composites for Their Efficiency in the Degradation of Antibiotics and Water Splitting

**DOI:** 10.3390/molecules30183712

**Published:** 2025-09-12

**Authors:** Syed Irfan, Sadaf Bashir Khan, Sheikha Lardhi, S. AlFaify

**Affiliations:** 1 State Key Laboratory of Environment-Friendly Energy Materials, Southwest University of Science and Technology, Mianyang 621010, China; syedirfan@swust.edu.cn; 2 School of Manufacturing Science and Engineering, Key Laboratory of Testing Technology for Manufacturing Process, Ministry of Education, Southwest University of Science and Technology, Mianyang 621010, China; sadafbashirkhan@swust.edu.cn; 3 Center for Integrative Petroleum Research, King Fahd University of Petroleum and Minerals, Dhahran 31261, Saudi Arabia; 4 Information and Computer Science Department, King Fahd University of Petroleum and Minerals, Al Khobar 31261, Saudi Arabia; 5 Advanced Functional Materials & Optoelectronic Laboratory (AFMOL), Department of Physics, Faculty of Science, King Khalid University, Abha 61421, Saudi Arabia; saalfaify@kku.edu.sa

**Keywords:** MXene, composites, antibiotics, HER, OER

## Abstract

The increasing occurrence of antibiotics in water bodies all over the world has raised concerns because of the prospect that they might have genotoxic and antibiotic-resistant consequences in both people and aquatic creatures. In particular, it has been discovered that the construction of hybrid photocatalytic composite materials has greater antibiotic degradation efficiencies. The hybrid photocatalysts deliver improved photoabsorbance, charge separation, transfer, and redox characteristics, as well as enhanced photostability and rapid recovery, due to their optimal characteristic qualities, including superior structural, surface, and interfacial properties. Additionally, metal-based electrocatalysts have garnered notable attention in the field of water splitting as they are low-cost, standard and have the potential to be used in green and clean technology. MXene, a family of two-dimensional transition metal carbides and nitrides, was discovered in 2011 due to its high conductivity, large surface area, and abundance of catalytically active sites. By making hybrid structures of MXene with other materials, which have shown better electrocatalytic activity than pure MXenes. The two half-cell processes involved in water electrolysis are the oxygen generation at the anode site and the hydrogen production at the cathode site. This review paper provides a summary of the latest advancements in the design of several hybrid systems, catalysts and their effectiveness in degrading a range of newly discovered antibiotic pharmaceutical pollutants in aquatic settings, as well as recent developments on the use of MXenes and MXene-based hybrid structures such as OER, HER, and bifunctional electrocatalysts for general water splitting.

## 1. Introduction

Growing crises that threaten the balance of the environment, human well-being, and economic resilience highlight the worldwide environmental and energy challenges of 2025. With 2023 officially being the warmest year ever measured and global CO_2_ concentrations surpassing 420 parts per million, climate change continues to pose the greatest threat, pushing global temperatures up 1.6 degrees Celsius above pre-industrial levels [1]. Storms, droughts, and wildfires are examples of extreme weather events intensified by global warming, which is expected to have resulted in $300 billion in damages in 2024 [2]. With estimates indicating that 5–10% of major urban centers may be flooded by 2030, coastal areas such as Miami and Shanghai are at risk due to rising sea levels caused by glacier melt and thermal expansion [3]. Despite a 3% increase in coal use in emerging countries due to energy security concerns, natural gas, which accounts for 75% of global greenhouse gas emissions [4], continues to dominate energy systems. These issues are exacerbated by pollution, as only 9% of plastics are successfully recycled each year, despite the annual output reaching over 380 million tons [5].

Pharmaceutical-activated chemicals are the most significant class of emerging aquatic pollutants, and their emergence has garnered considerable professional and public awareness in recent times. Due to their persistence and adverse effects on marine life, pharmaceutical chemicals are continually escaping into the environment, causing negative impacts [6,7]. Pharmaceutical substances that are biologically effective in the human system and have the prospect to exert significant effects on the neurological, immunological, circulatory, and hormonal systems are particularly concerning pollutants [8,9]. River water, irrigation water, and household wastewater all contain the medication carbamazepine (CBZ), which is even impermeable to microbial biodegradation [10]. As a psychotropic and antiepileptic drug, carbamazepine is an extensively used pharmaceutical that poses a risk to the atmosphere and biodegrades slowly in water [11]. To eliminate these antibiotics from the aquatic environment, photocatalysis has become a focus of study, as it is a clean, renewable technology with significant potential for tackling economic and environmental issues [12,13,14,15]. Photo-elimination may utilize sunlight to oxidize or degrade pollutants and trigger electron excitation transfer in a semiconductor photocatalyst [16,17,18,19]. Numerous semiconductors, sulfides, and metal oxides have been used to generate nanostructured compounds that serve as photocatalytic examples when exposed to sunlight [20]. However, single-material photocatalysts are unsuccessful due to the rapid recombination of photo-induced charge transfer, which results in low catalyst efficiency. Thus, a range of methods, including metal or non-metal doping, facet regulation, surface sensitization, and heterojunction creation, have been employed to enhance photocatalytic activity and reduce recombination [20,21,22,23,24].

The hydrogen energy is the most faultless clean energy currently available. It is widely used in the transportation, chemical, and aerospace sectors due to its high calorific value and low weight [25,26,27,28,29]. Water resources comprise a lot of hydrogen. Thus, it is almost essential to investigate technologies for the operational mass synthesis of hydrogen from water [30,31,32,33,34]. Water separates into hydrogen and oxygen when electricity is switched on. At the cathode and anode, respectively, the hydrogen generation reaction and the oxygen production reaction occur. Two electrons are transported during the heterogeneous catalytic procedure known as the hydrogen evolution reaction (HER). Regardless of the reaction medium, HER’s theoretical decomposition potential remains constant. However, distinct HER routes are determined by the reaction medium’s pH value, which has a significant influence on the reaction rate. For the HER, Pt is the perfect catalyst. However, it is not feasible to use on an extensive scale due to Pt’s high cost and incomplete deposits [35,36]. As a result, just 4% of industrial hydrogen generation is now formed using electrolytic water technology. As a result, research on HER catalysts is focused on either creating low-cost and effective non-noble metal catalysts or reducing the dose of noble metals.

When photons are absorbed, electron-hole pairs are created within the material’s bandgap, which starts the process of photocatalytic water splitting. According to the foundational study, these charge carriers then go to the catalyst surface, where they can initiate redox reactions with water, as long as the potential difference is greater than the 1.23 eV threshold. The first known example of water splitting into hydrogen and oxygen was recorded in 1972 [37], showing that the reaction could be triggered by visible light alone without the requirement for an external voltage. In 1980, the use of powdered TiO_2_ and SrTiO_3_ photocatalysts for UV-induced water splitting was first reported [38]. Since then, numerous studies have been conducted on the use of TiO_2_ as a photocatalyst in environmental remediation and water treatment [39,40]. TiO_2_ has been the subject of the majority of photocatalysis research, with the most significant amount of attention being paid to this material to the extent that TiO_2_ is frequently regarded as a prototype for transition metal oxide surfaces [41,42]. An organic framework that can perform photocatalysis without the aid of co-catalysts was recently reported by Du et al. [43]. When exposed to visible light, the framework demonstrated hydrogen evolution rates of 15,480 μmol/g.h from saltwater and 22,450 μmol/g.h from pure water. One approach that is seen as promising for meeting the industrial demands of hydrogen production on a larger scale is photocatalytic water splitting. In addition to facilitating effective chemical processes at room temperature when exposed to sunlight, photocatalysis has garnered significant interest due to its potential to create ideal technologies that transform abundant, harmless, and clean solar energy into chemical and electrical energy [44].

The oxygen evolution reaction (OER) occurs concurrently at the anode. Compared to the HER, which requires a larger overpotential to drive, the OER exhibits slower dynamics because it is a heterogeneous catalytic procedure that involves the transfer of four electrons. During water electrolysis, the OER is the phase that uses the most energy. The primary purpose of the overpotential needed for water electrolysis is to overcome the OER’s reaction energy barrier [36,45]. Precious metals like Ru and Ir, however, are expensive and challenging to use extensively. Thus, the formation of affordable and effective substitute catalysts is essential to OER catalyst research. Two-dimensional (2D) materials have gathered considerable attention in the catalysis community because of their distinct structure and electrical properties.

Over the last decade, 2D MXene materials have been widely utilized in various catalytic processes, including photoelectric, thermal, electrochemical, and photocatalytic applications [46,47,48,49]. MXenes have been extensively used in multiple fields in recent years due to their superior electrical, magnetic, chemical, and mechanical properties [50,51,52,53,54]. MXenes’ remarkable potential, which encompasses their high metallic conductivity, electronic properties, ease of functionality, and applications in sensors, energy storage, and environmental protection, makes them most suitable for a diverse range of applications. High surface area, biocompatibility, hydrophilicity, activated metallic hydroxide sites, zation, and tunable bandgap [51,55,56].

This review discusses the use of MXene composites, highlighting their flow, anti-fouling, and antibacterial properties in functional devices, such as catalyst membranes for continuous water treatment. The characterization methods (XRD, SEM, EDS) used to link structural characteristics to catalytic performance are covered. The paper concludes by outlining current constraints, including oxidation susceptibility, scalability issues, and restacking of MXene layers. It suggests future research avenues to overcome these obstacles and develop practical applications in green hydrogen production and wastewater treatment.

## 2. Mxene and Its Unique Characteristics

**Figure 1:**
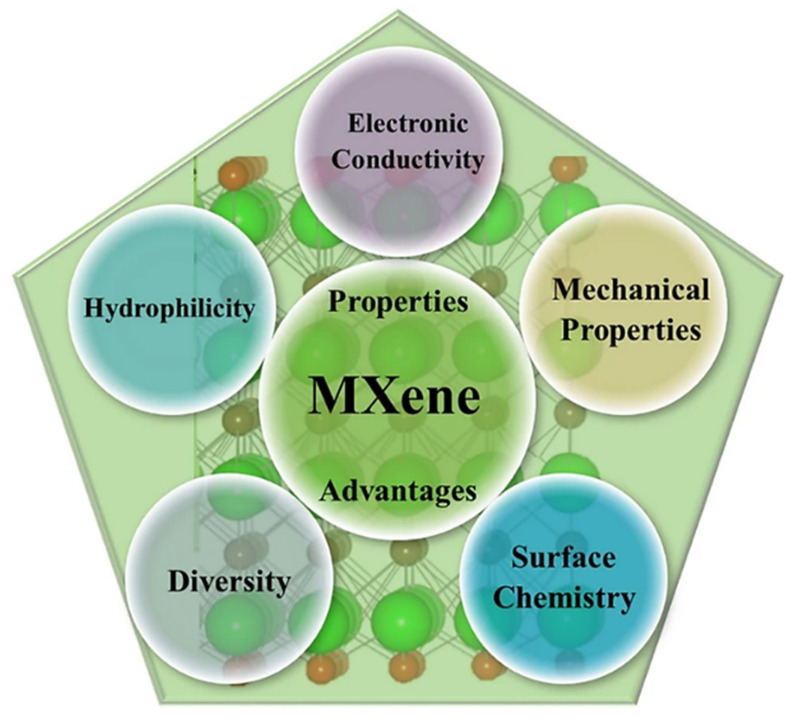
Unique characteristics of MXene materials. Reproduced with permission [59].

MXenes are a type of 2D material, mainly containing transition metal carbides and nitrides. They are usually obtained from layered ternary compounds called MAX phases, having the general formula M_n+1_AX_n_, Here M stands for early transitional metal (for example, Nb, Ti, Cr, V, Mo, etc.), A denotes the group 13 or 14 elements (such as Si, Al, Ga, etc.), while X is carbon and nitrogen [57]. The synthesis of MXenes requires selectively etching out layers of elements from the MAX phases, typically using fluorine-containing etchants, which yields ultra-thin layered nanosheets of the form M_n+1_X_n_T_x_, where T_x_ denotes surface terminations of hydroxyl (-OH) or oxygen (=O) groups, or fluorine (-F) groups [58]. These surface terminations are crucial in tuning the properties of MXenes, both chemically and physically, and thus, MXenes become highly adaptable for various applications. All the unique properties are shown in Figure 1.

One of the defining characteristics of MXenes is their outstanding electrical conductivity, which is comparable to metals. For example, some MXenes have conductivities as high as approximately 11,668 S/cm, making them the best candidates to use in energy conversion and storage, sensing, and electronics [60]. The reason they have such high conductivity is that metal layers are transferred to a 2D geometry, helping to transport electrons effectively. In addition, due to the structure of the MXene layers, they possess a significant specific surface area, providing multiple operating positions for chemical and adsorbent reactions [61]. This feature is especially beneficial in aspects of catalysis, supercapacitors, and batteries, where surface interactions dictate performance.

In addition to electrical properties, MXenes have significant tolerance and flexibility. The 2D panels of these materials are powerful yet flexible, allowing for their combination in composite materials or production into films without structural collapse. Another important property is thermal stability; MXenes retain their structure and function at high temperatures, thereby expanding their applications in severe operating conditions [62]. Additionally, MXenes have hydrophilic surfaces due to their functional groups, thereby increasing their dispersibility in water and their ability to interact with biological molecules, which is beneficial for biomedical applications. MXenes are a highly chemically adjustable material. By modifying the type of transition metal composition, carbon-to-nitrogen ratio, and surface termination, scientists can alter the structure of MXE and its electronic, optical, and catalytic properties. For instance, different metals or heteroatoms can be used to control conductivity or catalytic activity, while maintaining surface terminations alters hydrophilicity and chemical reactivity [63]. Such tunability enables the use of MXenes in a variety of applications, including energy storage systems (such as batteries and supercapacitors), environmental remediation, and biomedical technologies (such as drug delivery and antibacterial agents).

**Figure 2:**
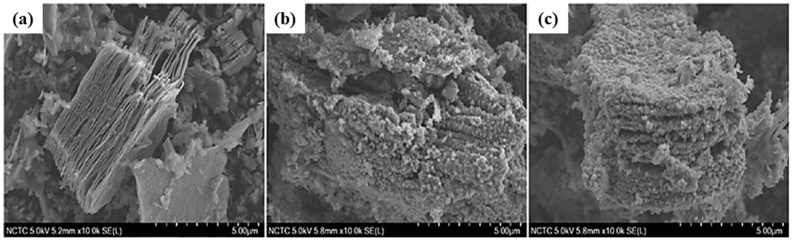
Scanning electron microscopy of (**a**) MXene, (**b**) MX-TiO_2_ (175 °C), and (**c**) MX-TiO_2_ (200 °C). Reproduced with permission [64].

**Figure 3:**
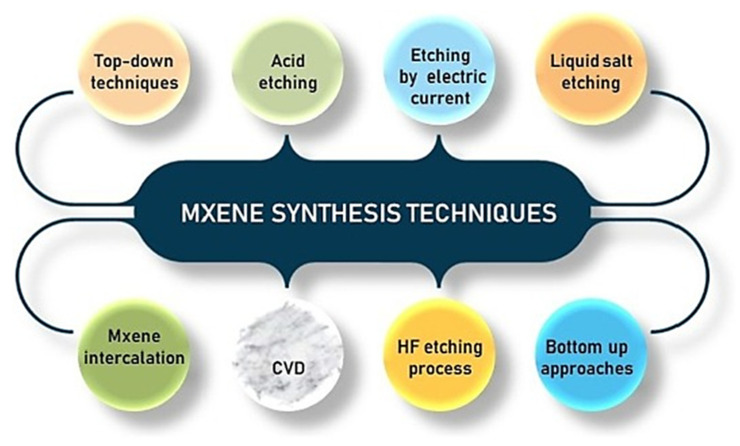
Different synthetic techniques of MXene materials. Reproduced with permission [74].

**Figure 4:**
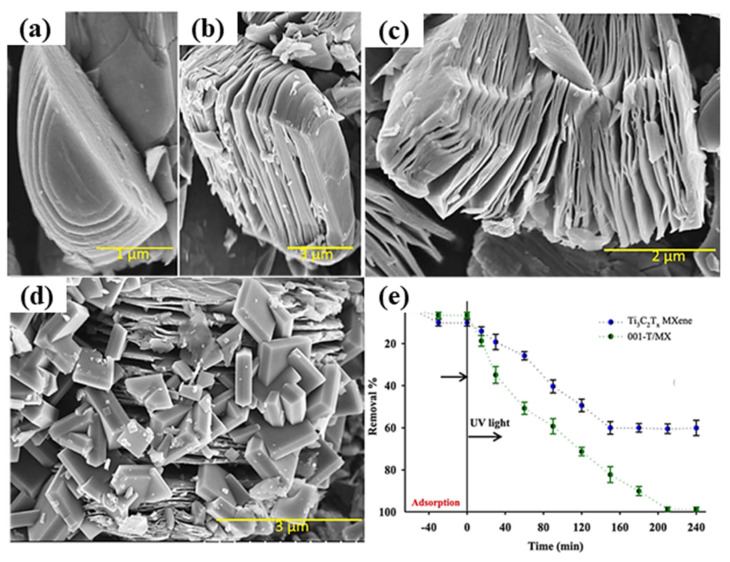
FE-SEM pictures of the (**a**) Ti_3_AlC_2_ MAX phase, (**b**) Ti_3_C_2_T_x_, (**c**) 001-T/MX photocatalyst, (**d**) after HF-30% etching, and (**e**) comparison of the CBZ photodegradation ability of 001-T/MX and Ti_3_C_2_T_x_ MXene photocatalysts. Reproduced with permission [65].

The SEM images of pure MXene and MX-TiO_2_ composites, prepared with and without NaCl at various hydrothermal temperatures. As shown in Figure 2, Pristine MXene exhibits a characteristic accordion-like layer structure. After hydrothermal treatment at 175 °C and 200 °C, several nanoparticles were detected to develop on the surface and between the layers of the MXene structure, as shown in Figure 2b,c [64]. The various synthesis techniques are shown in Figure 3. The morphological characteristics of Ti_3_AlC_2_, Ti_3_C_2_T_x_, and 001-T/MX were scrutinized using field-emission scanning electron microscopy. A compact, layered ternary carbide structure was perceived in Ti_3_AlC_2_. Following HF treatment, the compact symmetry of Ti_3_AlC_2_ transformed into a 2D structure, as shown in Figure 4d,e. When HF underwent an exothermic reaction, H_2_ gas was released, forming the accordion-like structure of MXene as shown in Figure 4b The SEM pictures, also shown in Figure 4c revealed the creation of heterostructures in nanosheets, with a significant percentage of the (001) planes of the anatase phase visible, and the emergence of (001)-TiO_2_ crystals in sheets. As anticipated, the Ti_3_C_2_T_x_ surface retained its hexagonal shape but became rough during the hydrothermal process. The achievement of the intended materials may be foreseen using the morphological characteristics of several material phases [65].

Despite the advantageous properties, functions, and applications of MXenes, several outstanding issues remain regarding their production and stability. Many MXenes’ properties have yet to be explored; some of their characteristics, such as ease of scalability, remain uncertain. Other synthesis techniques also use hazardous reagents, such as hydrofluoric acid, which poses safety and environmental hazards [66]. Furthermore, MXenes are susceptible to oxidation when exposed to air or liquids, which can deteriorate their performance over time. Current developments focus on creating safer and more affordable synthesis pathways, such as fluorine-free techniques and molten salt etching, in addition to surface modifications to enhance stability and prevent layer self-restacking, which can impede ion transit.

### 2.1. Structural Components

#### 2.1.1. Transition Metal Layers

The tightly packed layers of atoms make up the backbone of MXenes. Electronic and mechanical properties are determined by these transition metals (Groups 3–6). Titanium (Ti) is used in Ti_3_C_2_T_x_, the first MXene discovered, due to its excellent mechanical toughness and electrical conductivity (~11,000 S/cm) [67]. By introducing solid compounds or ordered double-metal structures, such as multi-metal MXenes like (Ti,V)_2_CT_x_ or (Mo_2_Sc)C_3_T_x_, customized features can be achieved, including improved catalytic efficiency and magnetic behavior.

#### 2.1.2. Carbon/Nitrogen Layers

Strong covalent and metallic connections are formed by the X atoms’ occupation of octahedral interstitial spaces between M layers. Recent developments include oxygen-containing MXenes (such as Ti_2_COT_x_), which replace some X sites to form oxy-carbides with adjustable band gaps. Still, these are generally C or N. Stability and reactivity are affected by the X composition; nitrides, such as Ti_4_N_3_T_x_, frequently show greater resistance to oxidation than carbides [63].

#### 2.1.3. Layer Thickness

The number of M layers stacked between X layers depends on the value of n. For instance, M_2_XT_x_ (such as Ti_2_CT_x_) is a single M layer that provides a high degree of flexibility. n = 3: Thicker sheets with increased mechanical strength (M_4_X_3_T_x_, for example, Ta_4_C_3_T_x_) [68]. Although increasing n tends to increase structural stability, it may decrease reaction surface accessibility.

#### 2.1.4. Surface Terminations

During synthesis, T_x_ groups are introduced by etching agents (such as HF and HCl/LiF). These terminations hinder restacking and modifying characteristics by capping exposed M atoms: O-O/-OH: Increase catalytic activity and hydrophilicity. O-F: May decrease conductivity but increase electrochemical stability. T_x_ may be precisely controlled by post-synthesis procedures (such as thermal annealing or chemical modification), which allows for property optimization for uses such as water splitting or antibiotic degradation [69].

#### 2.1.5. Layered Architecture

MXenes inherit a hexagonal close-packed structure based on MAX phases. Etching removes the parent MAX phase, yielding 2D sheets with an “accordion-like” morphology. Every layer is made up of n + 1 M layers intercalated between n X layers, capped with T_x_ groups on both termini. This structure offers a high surface-to-volume ratio (~300 m^2^/g) and numerous active sites, which are essential for adsorption and catalysis [70].

### 2.2. Synthetic Methods

Top-down etching techniques and bottom-up fabrication procedures are the two primary methods used to synthesize MXenes. The most well-known and popular technique is the top-down technique, which produces the 2D sheets of MXene (M_n+1_X_n_T_x_) by selectively eliminating the layer of “A” element from the original MAX phases (M_n+1_AX_n_) [71]. Usually, chemical etching procedures employing hydrofluoric acid (HF) or salts containing fluoride are used to accomplish this. The conventional top-down etching approach dissolves the “A” layers, such as silicon or aluminum, by directly applying strong HF acid to the powdered MAX phase [72]. The “A” atoms are eliminated by the HF etching reaction, which also functionalizes the exposed MXene surfaces with ending groups like -O, -OH, and -F, which affect the MXene’s characteristics. The quality, thickness of the layer, and surface chemical composition of the resultant MXenes are significantly impacted by parameters including HF temperature, concentration, and etching time [73]. However, because it is poisonous and corrosive, the direct use of HF raises serious safety and environmental concerns.

To overcome these limitations, modified acid etching techniques have been created that combine fluoride salts (such as LiF and NH_4_HF_2_) with hydrochloric acid (HCl) to produce HF in situ. By producing HF slowly in the reaction compound, this technique, often referred to as the “MILD” approach, reduces risks and improves the regulation of etching kinetics [75]. For instance, adding the MAX phase after breaking down LiF in HCl creates a safer etching condition while still effectively removing the “A” layers. After sonication, the in situ HF technique facilitates the delamination process. It creates few-layer or single-layer nanosheets by facilitating the intercalation of cations (such as Li^+^) between MXene layers. Systems that have been nitrogen-purged to reduce oxidation during synthesis are variations in this strategy [76].

Molten salt etching, another top-down method gaining popularity, involves heating MAX stages with molten fluoride salts, including NaF, LiF, and KF combinations at high temperatures, of approximately 550 °C [77]. Through chemical processes that result in volatile byproducts, such as AlCl_3_ gas, this technique quickly eliminates the “A” layers, leaving MXene layers that end with chlorine or other groups. Although the method eliminates liquid HF and permits quicker processing periods (minutes as opposed to hours), molten salt etching usually produces MXenes with lower crystallinity and more surface imperfections [78]. The MXene sheets must be purified and separated using post-synthesis rinsing and delamination procedures. Although it shows promise for large-scale production, this method consumes a significant amount of energy and requires optimization to enhance the quality of MXene.

In contrast to top-down approaches, the bottom-up formation of MXenes involves constructing MXene structures from smaller precursors atom by atom or molecule by molecule, often using solution-based crystal growth, molecular beam epitaxy, or chemical vapor deposition (CVD) [66]. The size, form, structure, and surface terminations of MXene can be exquisitely controlled through these approaches. For electrical and catalytic applications, bottom-up approaches can produce MXene films or heterogeneous structures with fewer defects and customized properties [79]. Unlike decreasing engraving, increasing production is still in the early stages for MXenes, and it faces the difficulties of such complicated precursors, prolonged processing times, and expansion issues.

## 3. MXene in the Degradation of Antibiotics

Concern about the presence of antibiotics in industrial discharges, drinking water, and wastewater has grown in recent years [80,81]. One of the most significant classes of synthetic antibiotics, fluoroquinolones (FQs), is widely used to treat diseases caused by a variety of species of bacteria [82,83]. Traditional sewage treatment measures cannot efficiently eliminate the majority of FQs due to their stable chemical structures and resistance to biological breakdown [84]. Thus, in aquatic environments, FQs have often been detected at detectable concentrations (for example, 0.6 to 5.6 μg/L) [85,86]. It has been revealed that environmental antibiotic residues can lead to the emergence of new, antibiotic-resistant bacteria, ultimately posing a threat to public health [87,88]. Therefore, new methods for giving FQs that remain in water are needed. FQs may now be eliminated from effluent using advanced oxidation procedures (AOPs) [89,90]. Photocatalytic expertise has garnered considerable attention as an AOP type due to its long-lasting stability and excellent efficiency. When exposed to UV light, a variety of conservative photocatalysts, such as TiO_2_, have shown the aptitude to break down these FQs by producing reactive oxygen species (ROS) [91,92]. The expansion of environmentally friendly and highly pH-tolerant sunlight/light-driven photocatalysts is necessary because the application of these photocatalysts has been hindered by their low light-harvesting ability, narrow pH range, difficulty in separation, and potential toxicity [93,94]. Both human health and aquatic ecology are seriously threatened by the presence of pharmaceuticals in marine environments [95,96].

Therefore, effective techniques are necessary to remove these harmful substances from water. An established and effective method for breaking down organic molecules is catalytic degradation. Pharmaceutical substances have been broken down using a variety of catalysts [97]. However, materials with improved catalytic activity are also sought after by researchers. In recent years, MXene-containing catalysts have also been shown to be an adequate substitute for conventional catalysts [98,99,100,101,102]. High surface area, a large number of surface terminations, semiconductor behavior with a tunable bandgap, and quick photogenerated electron-hole separation ability are the main characteristics of MXenes as co-catalysts [103]. These special qualities have made MXene-based photocatalysts a viable alternative to other 2D materials, such as graphitic carbon nitride (g-C_3_N) and transition metal dichalcogenides (TMDs).

### 3.1. Degradation of Antiepileptic Drug

The creation of a hybrid photocatalyst based on Ti_3_C_2_T_x_ nanosheets was achieved through an initial hydrothermal treatment procedure. The structural characteristics and morphology of the as-prepared photocatalyst, as well as the chemical makeup of MXene and its TiO_2_ derivatives in Ti_3_C_2_T_x_, represented as 001-T/MX, were systematically described. Through the Schottky circuit created between the TiO_2_-MXene surfaces, a controlled oxidation activity formed the heterojunction of the prepared photocatalyst. The pure MXene and the as-fabricated 001-T/MX nanohybrid for a drug of carbamazepine (CBZ) were scrutinized for their capacities in adsorption and photocatalytic degradation. The degradation measurements were significantly regulated in acidic conditions with a pH range of 3.0–5.0, and the measured Kapp value of CBZ under ultraviolet light was about 0.0304 per minute, greater than that under natural sunlight. The CBZ molecule was mixed by %OH and %O_2_ throughout the photocatalytic degradation process; specific degradation routes were proposed in response. For the CBZ breakdown, the new heterostructure 001-T/MX showed a lack of pertinence. Ti_3_AlC_2_, Ti_3_C_2_T_x_, and 001-T/MX morphological appearances were scrutinized using field-emission scanning electron microscopy. A compact, layered ternary carbide structure was obtained in Ti_3_AlC_2_. Following HF treatment, the compressed symmetry of Ti_3_AlC_2_ converted into a 2D structure, as shown in Figure 4a,b [104]. When HF underwent an exothermic reaction, H_2_ gas was released, forming according to the structure of MXene as shown in Figure 4c [105].

Figure 4d also shows the formation of heterostructures in nanosheets, with a noticeable percentage of the anatase phase planes visible, and the emergence of (001)-TiO_2_ crystals within the nanosheets. As anticipated, the Ti_3_C_2_T_x_ surface retained its hexagonal shape but became irregular during the hydrothermal process. The successful completion of the proposed materials may be predicted using the morphological characteristics of several material phases. Furthermore, the adsorption of CBZ onto the surface of the photocatalyst is considered an essential step before the photocatalytic degradation of CBZ [32]. Thus, within the reactor, in a dark situation, the adsorption capability of CBZ on the Ti_3_C_2_T_x_ and the 001-T/MX photocatalyst was examined. At around 5% and 7%, respectively, the adsorptive removal of pure MXene and 001-T/MX was very low, as shown in Figure 4e. Non-electrostatic interactions between the neutral CBZ and the negatively charged adsorbent may be the cause of the poor adsorption of CBZ. Since CBZ is a neutral molecule with a pKa (logarithmic value of the acidity constant) of 13.9, the pH of the system under study is unlikely to have an impact on the adsorption process [65].

Liquid chromatographytandem mass spectrometry (LC-MS/MS) was applied to recognize the primary intermediate products, thereby determining the breakdown routes of CBZ utilizing the 001-T/MX photocatalyst. Acridine, 2-aminobenzoic acid, 2-hydroxybenzoic acid, and formaldehyde acridine were recognized as the peaks. %OH free radicals produced from the photocatalyst’s heterostructure confronted the aromatic ring of CBZ, succeeding in intermediate and H-abstraction.

**Figure 5:**
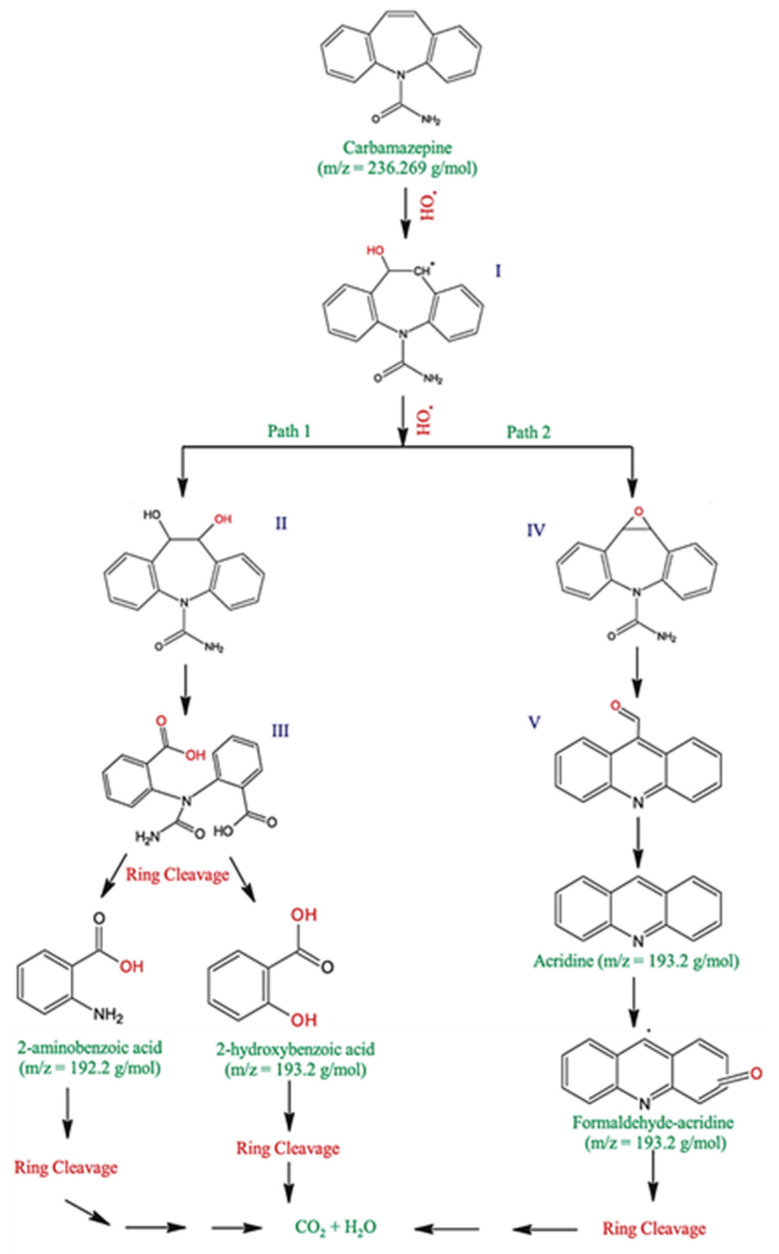
Potential paths for CBZ photocatalytic degradation by the 001-T/MX photocatalyst. Reproduced with permission [65].

Additionally, two distinct degradation pathways designated as route 1 and route 2, were selected based on the intermediates found, as shown in Figure 5 [106]. Formaldehyde-acridine, or intermediate D, was produced when acridine experienced another %OH substitution. Additionally, process 1’s additional transformation may provide benzoic acid (122 *m*/*z*) and aniline (93 *m*/*z*), which are then broken down into CO_2_ and H_2_O as a consequence of further oxidation and ring cleavage [107]. Accordingly, the ring breakage mechanisms in way 2 transformed the formaldehyde-acridine (intermediate D) into CO_2_ and H_2_O [65].

### 3.2. Degradation of Fluoroquinolone

Serious concerns have been raised by the presence of fluoroquinolones (FQs) in the atmosphere. This work examined the mechanism and photocatalytic degradation kinetics of ciprofloxacin (CIP) in ordered mesoporous g-C_3_N_4_ (ompg-C_3_N_4_). When exposed to sunshine radiation, ompg-C_3_N_4_ reacted to CIP deterioration 2.9 times faster than the bulk form of g-C_3_N_4_. This improvement might be clarified by ompg-C_3_N_4′_s high surface area and efficient charge carrier separation. The Langmuir-Hinshelwood kinetics model was employed to eliminate CIP, highlighting the significant role of surface reactions in the photocatalytic mechanism. Succeeding research on reactive species (RSs) using both ESR knowledge and RS scavenging studies has exhibited that the breakdown of CIP is instigated mainly by the photo hole (h^+^) and the consequent peroxide anion radical (O_2_^•^). The degradation ways of CIP were suggested based on the detection of mediates using liquid chromatography with tandem mass spectrometry and the estimation of reactive sites using Frontier Electron Densities. The degradation of FQs during the photocatalysis procedure was significantly affected by the piperazine mediator, as indicated by a comparison of the FQ degradation rates. The ompg-C_3_N_4_ demonstrated a highly desirable performance in decreasing antibiotic activity, as observed in an experiment on residual antibiotic activity. A ompg-C_3_N_4_ photocatalytic technique driven by sunlight may be effectively utilized to clean up the CIP-contaminated natural waterways, according to the tolerable photogenerated degradation of CIP in ambient water [108].

**Figure 6:**
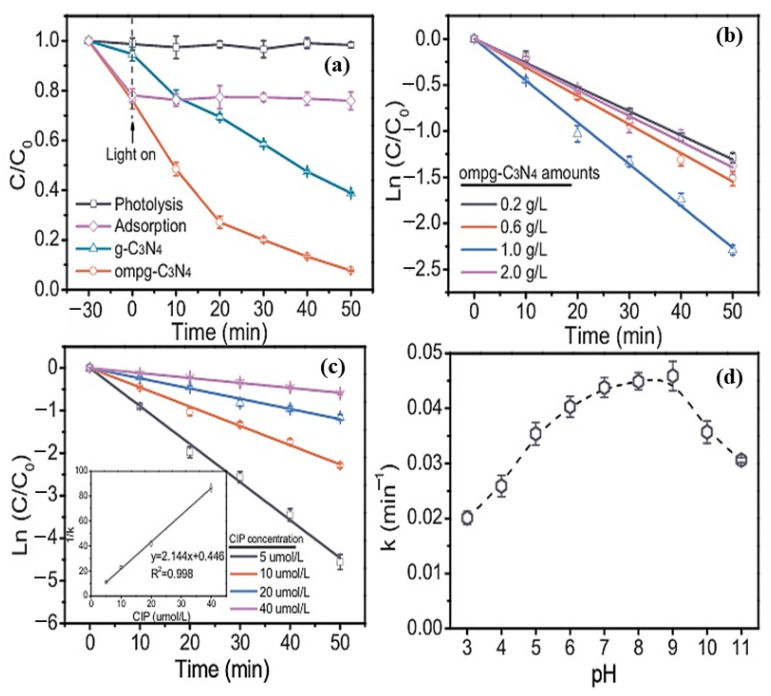
(**a**) Absorption, photocatalysis, and photolysis of CIP under simulated sunlight exposure are compared; (**b**) impact of ompg-C_3_N_4_ dose on CIP’s photocatalytic degradation kinetics, (**c**) impact of the initial concentration of CIP on the kinetics of its photocatalytic breakdown, and (**d**) CIP degradation kinetic rate constants at various pH levels. Reproduced with permission [108].

The concentration of CIP remained unchanged in the absence of the photocatalyst, as shown in Figure 6a, verifying the photostability of CIP during simulated exposure to sunshine. According to the adsorption experiment, under dark conditions, ompg-C_3_N_4_ absorbed 24.1% of CIP. In comparison, after 50 min of simulated sunshine irradiation, bulk C_3_N_4_ exhibited a degradation competence of 61.2%. Prominently, ompg-C_3_N_4_ significantly enhanced the degradation of CIP. For example, during the same period, 92.3% of CIP could be broken down by ompg-C_3_N_4_, which was around 2.9 times more than that of bulk g-C_3_N_4_. The impact of the ompg-C_3_N_4_ concentration on the photoinduced degradation of CIP is seen in Figure 6b. However, when the excess catalyst responds by scattering or reflecting photons to avert the photocatalyst’s excitation, subsequent increases in catalyst loading cause an undetectable decline in degradation efficiency.

Additionally, it was found that the number of active sites decreased when photocatalyst particles aggregated with increasing photocatalyst concentrations [109]. The influences of varying starting concentrations of CIP by a simulated sunlight ompg-C_3_N_4_ process are shown in Figure 6c. An increase in final substrate concentrations was found to cause the rate constants of CIP to drop. One description might be that the low breakdown rate of CIP was caused by larger concentrations of CIP absorbing incoming light, which decreased the photons needed to activate ompg-C_3_N_4_ [110].

As shown in Figure 6d, the impact of pH level on the ompg-C_3_N_4_-mediated photo-induced degradation of CIP was assessed. It was discovered that raising the pH caused the rate constants of CIP to increase. However, the degradation efficiency decreased when the pH increased further [108].

### 3.3. Degradation of Amoxicillin

Antimicrobial resistance caused by antibiotic misuse is a global problem that jeopardizes both human health and the ecosystem. Dingxin et al. described a photocatalytic method that is both operative and environmentally benign for breaking down the most commonly used antibiotic, amoxicillin. Better than the individual components, a special 2D/2D Bi_2_WO_6_/Ti_3_C_2_ MXene heterojunction with facet-to-face contact was carefully designed to enable total amoxicillin removal in less than 40 min. It was discovered that the composite photocatalyst enhanced the capacity for light adsorption, facilitated rapid electron-hole transfer, and exhibited improved photothermal adaptation characteristics, which are key components of its photocatalytic mechanism. These properties essentially facilitate the generation of reactive oxygen species, particularly photogenerated holes (h^+^) and superoxide anion radicals (•O_2_^−^). Advances in nanostructured catalyst design and photocatalytic methods are enabling innovative strategies for environmental remediation, including more effective wastewater treatment and the degradation of persistent organic contaminants [111].

**Figure 7:**
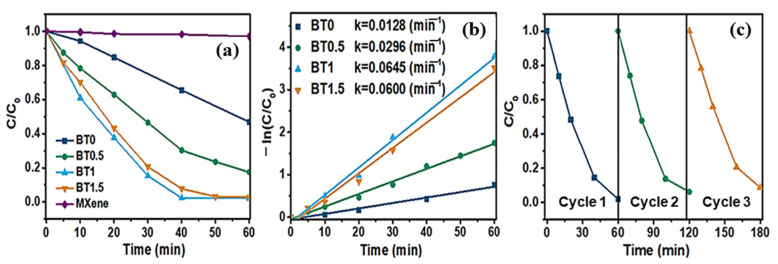
(**a**) Amoxicillin’s photodegradation and (**b**) the various samples’ degradation rate constants, and (**c**) Three amoxicillin degradation cycling tests over BT1. Reproduced with permission [111].

The samples were exposed to full-spectrum sun radiation for one hour to assess their amoxicillin-degrading competence. The BT0.5, BT1, and BT1.5 hybrids all display better photocatalytic activity against amoxicillin than bare Ti_3_C_2_ and bare Bi_2_WO_6_, as seen in Figure 7a. With over 100% amoxicillin degradation after 40 min, BT1 in particular has the most excellent photocatalytic activity of any sample and is on par with other photocatalysts that have been described. Since additional Ti_3_C_2_ speeds up electron transport during the photocatalytic process, growing the Ti_3_C_2_ level increases the rate of deterioration. The redundant Ti_3_C_2_, which partly hinders light absorption and protects the active sites on the Bi_2_WO_6_ surface, is the reason for the modest performance drop caused by an excessive loading quantity of 1.5%. The pseudo-first-order approximation, as shown in Figure 7b, is followed by all photocatalytic processes, where the introduction of Ti_3_C_2_ results in a rise in apparent rate constants (k).

**Figure 8:**
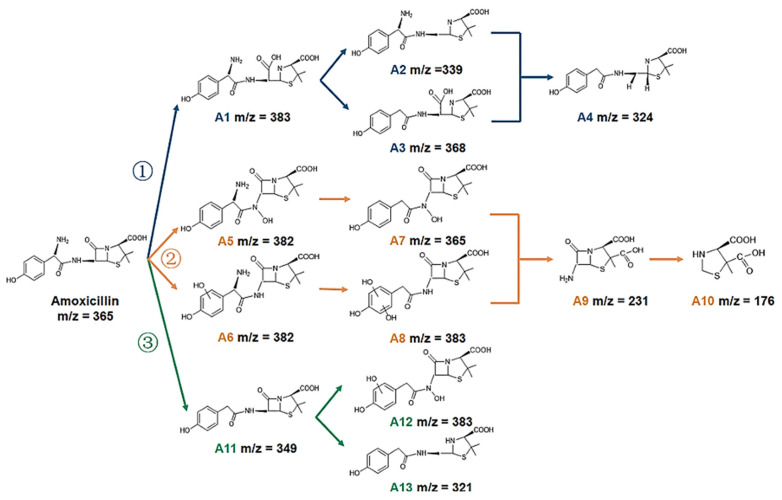
Amoxicillin’s suggested photodegradation paths across BT1. Reproduced with permission [111].

The greatest deterioration rate constants are found in BT1, which is five times more than that of pristine Bi_2_WO_6_. Furthermore, to investigate the stability of the synthesized hybrids, cyclic tests have been carried out under the same conditions, as illustrated in Figure 7c. After three successive loops, the degradation rate for BT1 remains unchanged, mainly, showing the hybrid catalysts’ outstanding durability and reusability for real-world environmental requests. The LC-MS analysis identified the intermediates produced during the BT1-driven photodegradation of amoxicillin. Based on these intermediates, three potential routes for the breakdown of amoxicillin are recommended, as shown in Figure 8. Blue arrows point to Pathway 1, where the β-lactam ring is first lipidated to produce amoxicillin penicillanic acid [111].

### 3.4. Degradation of Enrofloxacin

**Figure 9:**
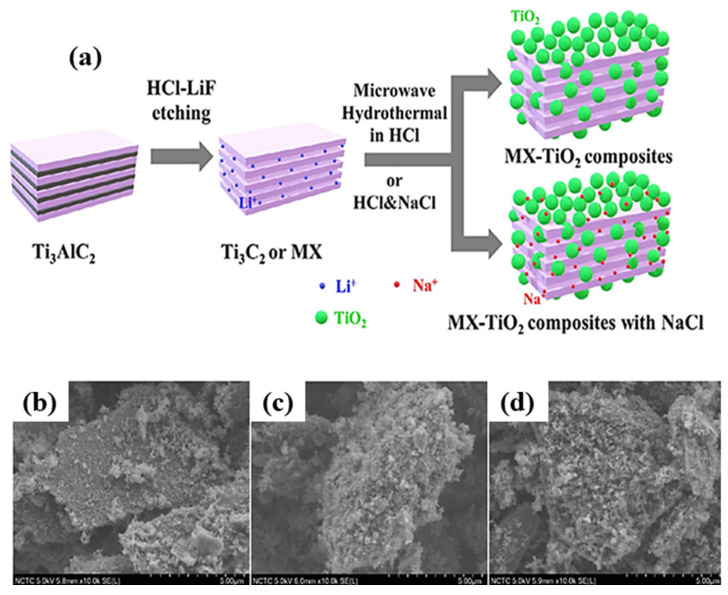
(**a**) Schematic representation of the production of Ti_3_C_2_-TiO_2_ (MX-TiO_2_) composites, with or without NaCl. SEM images of (**b**) MX-TiO_2_ at 175 °C/NaCl, (**c**) MX-TiO_2_ at 200 °C/NaCl, and (**d**) MX-TiO_2_ at 225 °C/NaCl. Reproduced with permission [64].

MXenes, a new type of two-dimensional transition metal carbides or nitrides, have gathered remarkable attention for a range of uses because of their special properties, which include ion intractability, hydrophilicity, and high electrical conductivity. Using a rapid and easy microwave hydrothermal method by applying heat treatment on the HCl/NaCl mixture solution, Ti_3_C_2_ MXene, or MX, is converted into MX-TiO_2_ composites. This process creates fine TiO_2_ nanoparticles on the M-X parent structure and gives the resultant MX-TiO_2_ composites photocatalytic activity, as shown in Figure 9a. The composites were used to remove the common contaminated antibiotic enrofloxacin (ENR) from water. By adjusting the temperature of hydrothermal, the proportion of M-X and TiO_2_ may be regulated, producing nanocomposites with adjustable adsorption and photocatalytic capabilities. Since composites made without NaCl were unable to adsorb enrofloxacin effectively, it was shown that the inclusion of NaCl had a significant impact. Sodium ions are concurrently intercalated into the nanocomposite structure when NaCl is added to the hydrothermal process, meaningfully increasing ENR adsorption from 1 to 6 mg ENR/g composite.

Additionally, it decreases the speed of the conversion of MX to TiO_2_, which results in a more uniform and smaller distribution of TiO_2_ particles on the structure. Even while composites without NaCl had a greater TiO_2_ content, MX-TiO_2_/NaCl nanocomposites, which had sodium intercalated in their structures, demonstrated stronger ENR adsorption and photocatalytic activity. High photocatalytic degradation efficiency results from the effective destruction of adsorbed ENR on the composites by free radicals produced by the photoexcited TiO_2_ particles. Sukidpaneenid et al. illustrated how adsorption and photocatalytic degradation of the produced chemicals work in concert [64]. The SEM of pure MX and MX-TiO_2_ composites made with and without NaCl at various hydrothermal temperatures are shown in Figure 9. As shown in Figure 9a, Pure MX exhibits a characteristic accordion-like layer structure. Several nanoparticles were seen to develop on the surface and between the layers of the MXene structure after hydrothermal treatment at 175 °C and 200 °C, as seen in Figure 9b,c. The particles that progress in systems with NaCl addition, Figure 9d seem to be smaller than those in the system without NaCl.

Even though they are now hardly discernible, the composites’ sheet-like and layered structure is still noticeable. During this hydrothermal process, the sodium cations have inhibited the development of the TiO_2_ particles on the MXene nanosheets, or NaCl, intercalated between the MXene layers. Several fine TiO_2_ particles were observed to form on the Ti_3_C_2_ nanosheets upon thermal oxidation in air [112] or solvothermal in liquid media of ethylene glycol, HF, and isopropyl alcohol [113]. These structures of the MX-TiO_2_ nanocomposite are reasonably similar to those reported in the literature.

**Figure 10:**
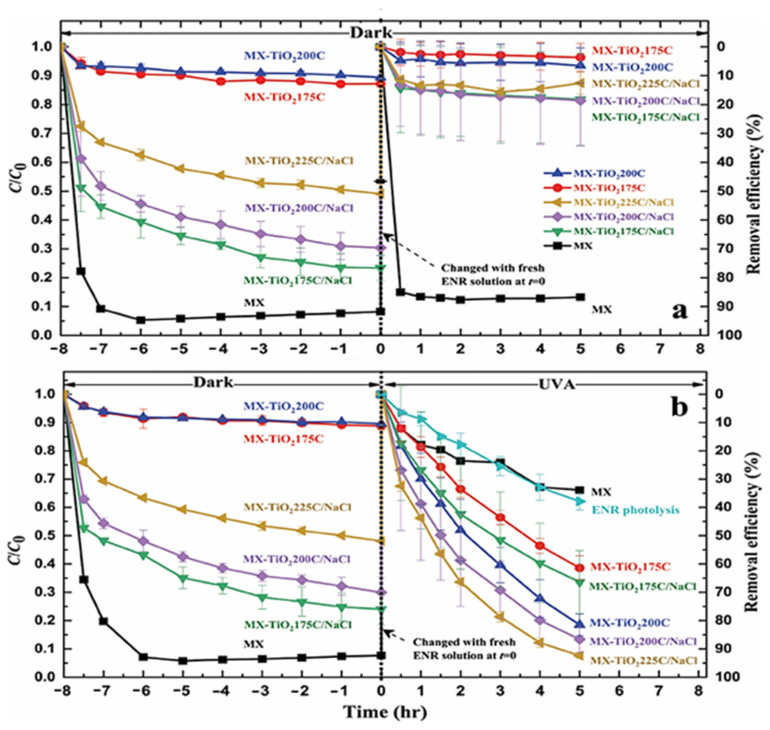
Enrofloxacin (ENR) removal by MX and MX-TiO_2_ composites as a function of time under (**a**) 8 + 5 h of dark conditions and (**b**) 8 h of dark conditions plus 5 h of UVA irradiation. Reproduced with permission [64].

The ENR was eliminated using MX and MX-TiO_2_ composites in a dark environment with UVA irradiation. After the materials had been incubated with ENR for eight hours in the dark, the UVA irradiation was carried out. Five hours later, the ENR solution was poured and replaced with a new ENR solution. The ENR elimination effectiveness in a dark environment is shown in Figure 10a. The tidy MX has a removal effectiveness of about 92% because of its excellent ability to absorb ENR. The tidy MX’s cation exchange is most probably the leading cause of this. It has been revealed that MX etched by LiF contains Li cations in its structure, which may help MX adsorb cations [114].

On the other hand, the MX-TiO_2_ that has been hydrothermally treated with 1 mol/L HCl has a minimal quantity of Li in its structure and can thus only remove 11.2% of the ENR. The sharp decline in the MX-TiO’s Li concentration. The effectiveness of ENR elimination under UVA irradiation is seen in Figure 10b. To characterize the ENR removal efficiency of the materials that is attributable to the UVA irradiation effect alone, the curves under UVA irradiation were previously normalized by the ENR removal efficiency under the dark conditions at the same respective time in Figure 10a. For comparison, the ENR photolysis under UVA was also shown. MX had very little UV photodegradation activity against ENR, as shown by the fact that its ENR removal effectiveness under UVA irradiation was comparable to the drop in ENR concentration brought on by photolysis itself [64].

### 3.5. Degradation of Norfloxacin

**Figure 11:**
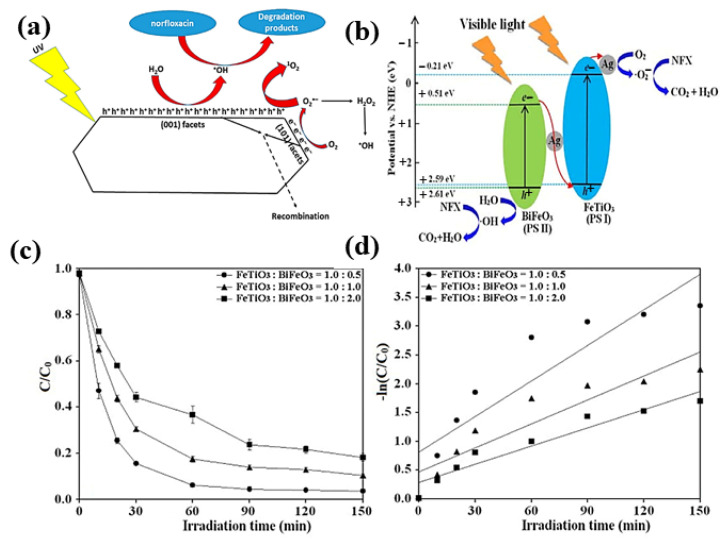
(**a**) Faceted TiO_2_/Ti film photocatalysis mechanism. Reproduced with permission [110]. (**b**) A potential Z-scheme mechanism diagram System Ag/FeTiO_3_/Ag/BiFeO_3_, (**c**) The impact of FeTiO_3_ and BiFeO_3_ mass ratios, and (**d**) the kinetics of degradation reactions on photocatalytic activity. Reproduced with permission [119].

It belongs to the class of fluoroquinolones, used to treat urinary tract infections [115]. Norfloxacin is now detected in wastewater at high concentrations, particularly in hospitals, and is considered a potential contaminant in the aquatic environment. Because of their extensive usage and environmental toxicity, fluoroquinolone antibiotics have caused a great deal of worry in recent years [115,116]. Numerous studies have shown the photocatalytic degradation of norfloxacin utilizing various materials [117]. The experimental results showed excellent photocatalytic performance toward the degradation of norfloxacin across multiple water matrices. Sayed et al. [118] prepared a novel immobilized TiO_2_/Ti film with exposed facets via a simple one-pot hydrothermal route to be used in the degradation of norfloxacin from aqueous media. It was observed that OH is primarily involved in the photocatalytic degradation of norfloxacin by faceted TiO_2_/Ti film, as shown in Figure 11a. Furthermore, Tang et al. [119] observed outstanding performance of visible light-driven photocatalysis for the sol–gel approach, followed by a photo reduction procedure to break down norfloxacin utilizing a unique Z-scheme Ag/FeTiO_3_/Ag/BiFeO_3_ as produced. When employing Ag/FeTiO_3_/Ag/BiFeO_3_ at 2.0 weight percent Ag, the findings show that the photocatalytic degradation extent reaches 96.5% after 150 min and can be reprocessed with outstanding photocatalytic stability, as shown in Figure 11b–d.

Additionally, Lv et al. [120] used a solvothermal technique to create copper-doped bismuth oxybromide and evaluated its capacity to break down norfloxacin in the presence of visible light. Cu-doped BiOBr, as prepared, demonstrated high activity in the photocatalytic degradation of norfloxacin under visible-light irradiation, with a photocatalytic degradation constant. This was due to its improved light-harvesting properties, enhanced charge separation, and interfacial charge transfer. Additionally, it retained 95% of its initial activity even after five continuous catalytic cycles. Similarly, Tang et al. [121] proposed Bi_2_WO_6_, another bismuth-based catalyst, and used it to photodegrade norfloxacin in a nonionic surfactant Triton-X100 dispersion when exposed to visible light. The findings showed that adding TX100 might significantly increase the breakdown of scarcely soluble norfloxacin. At the critical micelle concentration, TX100 stimulated norfloxacin photodegradation and was highly adsorbed on the Bi_2_WO_6_ surface. The degradation of antibiotics by MXene based materials are summarized in Table 1. 

**Table 1:** Degradation of antibiotics by MXene-based material.

MXene-Based Membranes	Mechanism	Antibiotics	Reference
Ti_3_C_2_T_x_/CNFs	Electrostatic repulsion/sieving	azithromycin	[122]
Ti_3_C_2_T_x_	Electrostatic repulsion/molecular sieving	Erythromycin	[123]
Ti_3_C_2_T_x_	Electrostatic repulsion/molecular sieving	Penicillin	[123]
Ti_3_C_2_T_x_	Electrostatic repulsion/molecular sieving	Rifampicin	[123]
Ti_3_C_2_T_x_	Electrostatic repulsion/molecular sieving	Bacitracin	[123]
Ti_3_C_2_T_x_	Photocatalytic inactivation	*E. coli*	[124]
g-C_3_N_4_@MXene	Electrostatic repulsion/molecular sieving	Tetracycline hydrochloride	[125]
g-C_3_N_4_/TiO_2_/kaolinite	Chemical stripping and self-assembly	ciprofloxacin	[126]
g-C_3_N_4_/C-TiO_2_	S-scheme heterojunction	ciprofloxacin	[127]
sepiolite/g-C_3_N_4_/Pd	Photocatalytic mechanism	ciprofloxacin	[128]
Au@ZnONPs-rGO-gC_3_N_4_	photodegradation mechanism	Ciprofloxacin, Levofloxacin	[129]

## 4. MXene in Water Splitting

Electrocatalytic water splitting holds considerable promise as a sustainable and eco-friendly method for producing hydrogen and oxygen, with the latter serving as a renewable alternative to conventional fossil fuels. As an electrocatalyst for water splitting, MXene, a unique two-dimensional (2D) layered family of materials, has drawn a lot of interest. This adaptable substance can be modified to enhance its stability and electroactive surface properties for improved electrocatalytic activity [130]. The development of inexpensive, non-noble metal-based electrocatalysts for water splitting has garnered significant interest due to their potential to provide clean, green hydrogen fuel. A family of two-dimensional transition metal nitrides, carbides, and carbonitrides was discovered in 2011. Because of their high electrical conductivity, huge surface area, and abundance of catalytically active sites, these metals have shown potential performance as electrocatalysts in the water splitting process. However, the agglomeration and restacking of MXene flakes restrict their long-term stability and recyclability. By mixing MXene with other materials to generate hybrid structures, which have shown better electrocatalytic activity than pure MXenes, this issue may be addressed. The two half-cell processes involved in electrolysis of water are the OER at the anode and the HER at the cathode [131].

### 4.1. Hydrogen Evolution Reaction

**Figure 12:**
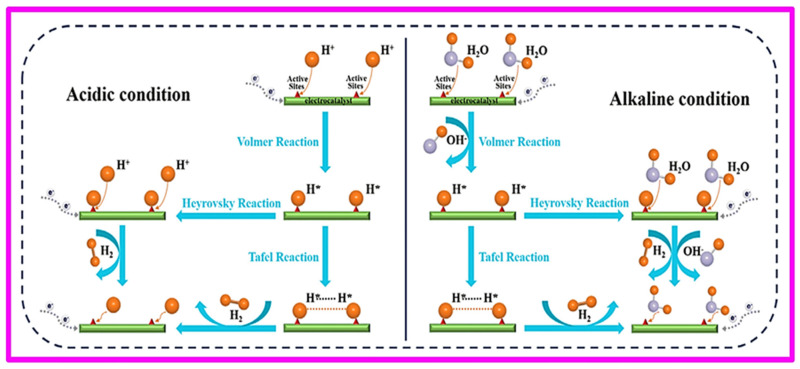
HER schematics in alkaline and acidic environments. Reproduced with permission [137].

Because of its immediate use in a variety of sectors, including water electrolysis, and relative simplicity, the hydrogen evolution process HER is one of the electrochemical reactions that has been considered the most. The HER exhibits noticeably quicker kinetics when it takes place on noble metal electrodes, especially those made of platinum group metals (PGM), as opposed to the sluggish kinetics seen in the OER and oxygen reduction reaction (ORR). Practical current densities may be achieved at a few tens of millivolts of overpotential due to these improved kinetics [132,133]. Inquiries into the mechanism of HER on metallic surfaces began in the early 1950s and were primarily focused on nickel [132]. Two electrons are transferred during the two-step hydrogen evolution reaction at the cathode, which also includes the adsorption and desorption of intermediate molecules. The Volmer step is the first stage of HER in an acidic environment, where hydrogen is reduced by one electron and then adsorbed at the cathode surface (H*) [134,135]. Depending on the quantity of adsorbed hydrogen atoms, there are two possible routes for the production of hydrogen molecules after the preceding step. When the H* atom’s concentration is very low, it takes an electron and a proton from the water, which causes H_2_ to desorb from the cathode surface. The sequence is known as the Volmer–Heyrovsky mechanism, and the process is known as the “Heyrovsky step.” Additionally, when the amount of H* is considerable, two nearby H* atoms on the cathode’s surface combine to form a hydrogen molecule. According to HER diagrams in acidic and alkaline environments, this process is referred to as the “Tafel step,” and the sequence of stages is known as the Volmer–Tafel mechanism, as shown in Figure 12 [132,136,137], which shows the schematics of HER in acidic and alkaline environments. The primary mechanism of the HER for different catalysts can often be identified by comparing the Tafel slope values [138].

#### MXene-Based Nanocomposites for HER

**Figure 13:**
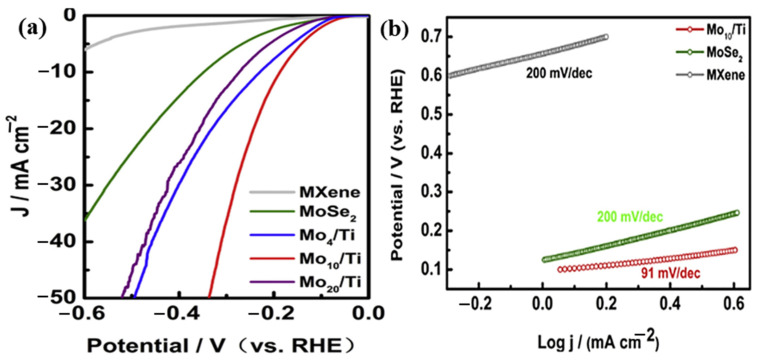
(**a**) LSV curves and (**b**) Tafel plots of Ti_3_C_2_ MXene, MoSe_2_, and various MoSe_2_/MXene hybrid nanoflowers. Reproduced with the permission [140].

The surface shape of MXenes has a significant impact on their electrocatalytic activity. Yuan et al. [139] used a two-step procedure that included hydrolyzing bulk MAX ceramics and then HF etching to create an electrocatalyst based on highly active MXene nanofibers with a sizable specific surface area. MXene nanofibers have a Tafel slope of 97 mV/dec and a low overpotential of 169 mV at 10 mA/cm^2^, which are noticeably greater than those of Ti_3_C_2_ flakes. Ti_3_C_2_ Mxene nanofibers perform better than conventional MXene nanosheets because of their larger specific surface area, which exposes more active spots on their cross-section. Jia-Jun et al. effectively synthesized 3D-MoSe_2_@Ti-MXene nanoflowers with exclusive nanoscale petal morphologies using a simple hydrothermal technique. The synthesized MoSe_2_@Ti-MXene’s electrocatalytic properties in a 0.5 M H_2_SO_4_ solution for HER were examined. According to Figure 13a, which compares the electrocatalytic performance of Ti_3_C_2_ MXene and MoSe_2_ as well as various hybrid MoSe_2_/MXene nanoflowers, the MoSe_2_@Ti-MXene hybrid performed noticeably better than bare MoSe_2_. It showed an overpotential of 180 mV and the lowest onset potential, at 61 mV, at 10 mA/cm^2^. In comparison, the bare MoSe_2_ needed an overpotential of 300 mV to achieve the same current density of 10 mA/cm^2^. A slower charge transfer rate is shown by the Tafel slope of 200 mV/dec for bare MoSe_2_, as seen in Figure 13b. The Tafel slope, however, was significantly dropped to only 91 mV/dec for the MoSe_2_@Ti-MXene composite, suggesting a quicker charge transfer between MoSe_2_ and Ti-MXene. The study’s findings indicate that the hybrid structure’s enhanced electrocatalytic performance is a result of its increased conductivity and effective electron transport [140].

Reghunath and his co-researchers have formed and evaluated a flexible hierarchical composite of Bismuth ferrite/chromium carbide as BiFeO_3_/Cr_2_CT_x_ MXene as an electrocatalyst for water splitting. This strategy suggested a simple way to create Cr_2_CT_x_ MXene from the chromium aluminum carbide MAX Phase. The elimination of aluminum atomic layers from the Cr_2_AlC MAX structure was confirmed by high-resolution transmission electron microscopy, scanning electron microscopy, and X-ray diffraction investigations. With a low overpotential of 128 mV in 1 M KOH, electrochemical experiments showed that the BiFeO_3_/Cr_2_CT_x_ MXene composite, which was made with a decreased Al_2_O_3_ composition, performed very well in the hydrogen evolution process. The Tafel slope and charge transfer resistance have been calculated to be 53.3 mV/dec and 0.16Ω, respectively. The BiFeO_3_/Cr_2_CT_x_ MXene electrode used only 1.81 V of cell potential in a di-electrode electrolysis system to generate 10 mA/cm^2^ with sustained stability. This work proposed a simple method for creating Cr_2_CT_x_ MXene composites for HER applications while highlighting the possible use of BiFeO_3_/Cr_2_CT_x_ MXene in HER [141].

The electrical characteristics of the various transition metal components prejudice the HER activity of MXenes. According to Zhi et al., Mo_2_CT_x_ MXenes have superior HER activity compared to Ti_2_CT_x_ and may be used as an active and stable catalyst for HER in acid [142]. In this study, Mo_2_Ga_2_C and Ti_2_AlC are used as raw materials for HF etching, which yields Mo_2_CT_x_ and Ti_2_CT_x_ MXene, respectively. At 10 mAcm^2^, Mo_2_CTX had a lower initial overpotential of 283 mV than Ti_2_CT_X,_ at 609 mV. The catalyst was unstable, as shown by the additional reduction in Ti_2_CT_x_’s HER activity during continuous cycling. After around 30 cycles, Mo_2_CT_x_ achieves an overpotential of 305 mV at 10 mA/cm^2^, exhibiting only a modest initial drop in HER activity as compared to Ti_2_CT_x_, Mo_1.33_CT_x,_ and Mo_2_CT_x,_ which are two Mo-based MXenes that were created by Intikhab et al. [143]. They discovered that.

In contrast, operational durability had little to no effect on HER activity; stoichiometry and atomic surface structure had a significant influence. The HER activity of Mo_1.33_CT_x_ is lower than that of Mo_2_CT_x_. The materials’ over potentials are 422 and 239 mV at 10 mA/cm^2^, respectively. The variance in surface electronic characteristics and the loss of hcp sites with a core six-fold coordinated carbon atom, which lowers the fraction of the ideal -O terminated, are the causes of Mo_1.33_CT_x_ poor HER activity. These discoveries have the potential to be applied to other technology-related electrochemical processes and provide essential insights into the creation and advancement of molybdenum-based 2D materials [144,145,146].

In the HER process, the conductive Cr_2_C and Cr_2_CO_2_ are advantageous for electron transport [147]. Transition metal modification or hydrogen coverage may modify the free energy of hydrogen adsorption. The creation of carbon vacancy engineering for Cr_2_CO_2_ MXene may also improve HER results. The preparation of 2D Cr-based MXene for potential electrocatalytic water splitting is made possible by these first findings. A new structure of MXene nanofibers as Ti_3_C_2_ NFs was created by Yuan et al. [139], and the nanofiber morphology displayed a thickness of 40–60 nm. Additionally, the MXene NFs synthetic approach can be expanded to include the synthesis of other MXenes and applied to promising applications, such as catalysis, batteries, and supercapacitors. Nearly every prior study up to this point has referred to MXene as metal. But according to Abdoulaye et al., the 2D-Ti_3_N_4_ MXene has both semiconductor and metallic characteristics [148]. Additionally, this work demonstrates the occurrence of T_x_ = O and/or OH, which is susceptible to surface oxidation, and offers an integrated structural characterization of exfoliated Ti_4_N_3_T_x_ MXene. With a Tafel slope of around 0.19 V/dec and an over potential of 0.3 V at 10 mA/cm^2^, the exfoliated Ti_4_N_3_T_x_ shows excellent HER performance.

**Figure 14:**
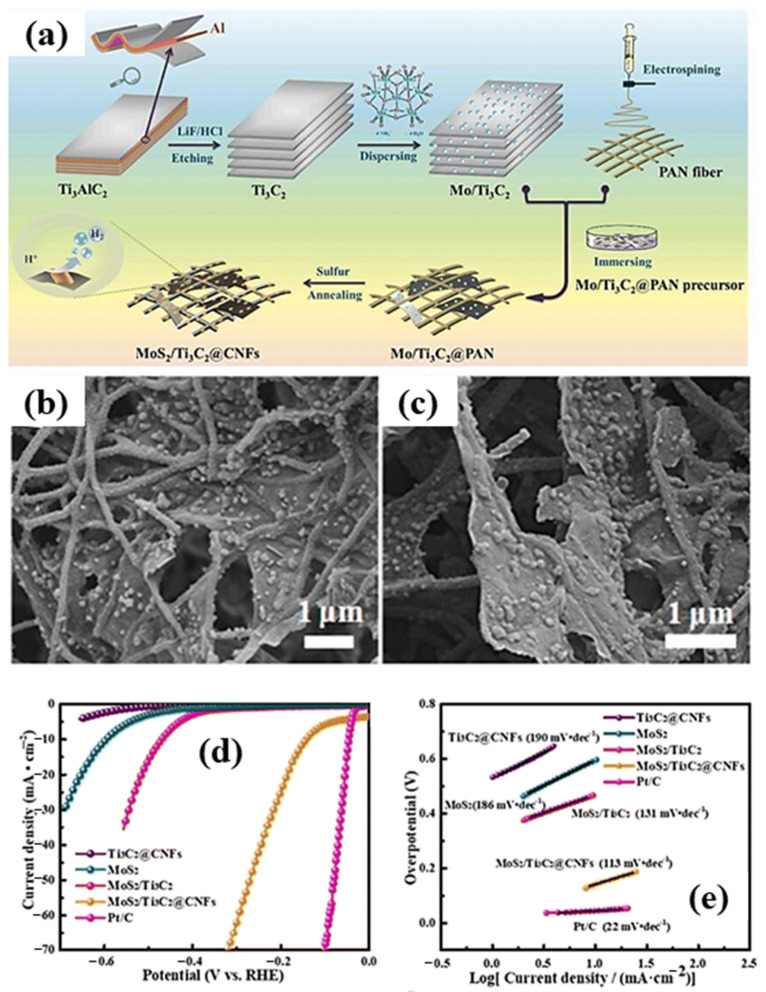
(**a**) MoS_2_/Ti_3_C_2_@CNFs synthetic schematic. MoS_2_/Ti_3_C_2_@CNFs SEM pictures at varying magnifications; (**b**) 12,000×, (**c**) 1800×, (**d**) the polarization curves of MoS_2_, MoS_2_/Ti_3_C_2_, Ti_3_C_2_@CNFs, MoS_2_/Ti_3_C_2_@CNFs, and Pt/C catalysts, and (**e**) Tafel plots of several catalysts. Reproduced with permission [149].

With its hexagonal structure and sandwich-like arrangement of sulfur and molybdenum atoms, molybdenum disulfide (MoS_2_) is a well-known member of the transition metal dichalcogenides (TMDCs) family. Using MoS_2_, Ti_3_C_2_, and carbon nanofibers (CNFs), Ma et al. created a very effective and stable HER electrocatalyst, as shown in Figure 14a. Ti_3_C_2_ and CNFs were used to develop a plane-like skeletal structure in the first stage, which helped to stop the MXene flakes from restacking. A stereostructured MoS_2_/Ti_3_C_2_@CNFs was then achieved by the spontaneous development of MoS_2_ on the fiber skeleton. Multiple magnification SEM images of MoS_2_/Ti_3_C_2_@CNFs are shown in Figure 14b,c, respectively. This special structure led to a notable increase in electrical conductivity and a more extensible layered structure.

Figure 14d,e displays the HER polarization curves for the catalysts MoS_2_, MoS_2_/Ti_3_C_2_@CNFs, MoS_2_/Ti_3_C_2_, and Ti_3_C_2_@CNFs in a 0.5 M sulfuric acid electrolyte and Tafel plots of different catalysts, respectively. For comparison, the catalytic performance of commercial Pt/C was also examined. At a HER current of 10 mA/cm^2^, the MoS_2_/Ti_3_C_2_@CNFs catalyst demonstrated a much-reduced over potential of 142 mV vs. RHE in contrast to 471 mV for MoS_2_/Ti_3_C_2_ and 596 mV for MoS_2_ alone. This suggests that the fiber skeleton structure of MoS_2_/Ti_3_C_2_@CNFs significantly enhanced the HER’s catalytic performance, demonstrating that CNFs successfully gave the catalyst a solid framework that enabled efficient MoS_2_ loading and demonstrated outstanding HER activity. Moreover, the HER performance of Mxene based materials are summarized in Table 2.

**Table 2:** HER performance of MXene-based materials as electrocatalysts.

Electrocatalyst	Electrolyte	Tafel Slope	Overpotential	References
Ti_2_CT_x_	0.5M H_2_SO_4_	100 mV/dec	75 mV	[150]
Ti_3_C_2_T_x_	0.5M H_2_SO_4_	97 mV/dec	169 mV	[150]
Pd/Ti_3_C_2_T_x_–CNT	0.1M KOH	50 mV/dec	158 mV	[151]
Ru@Ti_3_C_2_T_x_-Vc	1M KOH	32 mV/dec	35 mV	[152]
Pt/Ti_3_C_2_T_x_	0.5M H_2_SO_4_	29.7	34	[153]
Ti_3_CN(OH)x@MoS_2_	0.5M H_2_SO_4_	64	120	[154]
Rh–CoNi LDH/MXene	1.0M KOH	43.9	74.6	[155]
Co-NCNT/Ti_3_C_2_T_x_	1.0M KOH	78	190	[156]
BiFeO_3_/Cr_2_CT_x_	1.0 M KOH	53.3	128	[141]
WS_2_@MXene/GO	1.0M KOH	58	43	[157]
Ti_3_C_2_T_x_: Co	1.0M KOH	103.3	103.6	[158]

### 4.2. Oxygen Evolution Reaction

**Figure 15:**
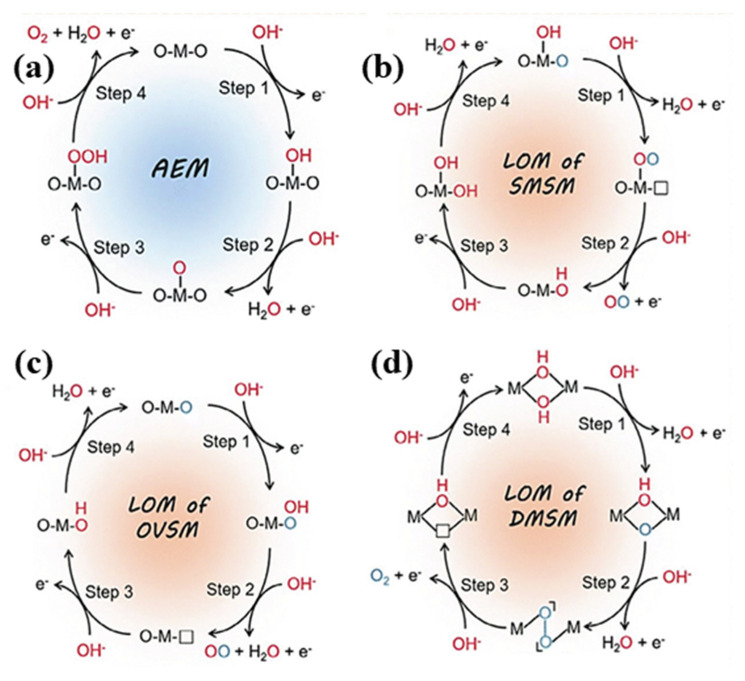
The overall OER’s fundamental mechanism in which (**a**) AEM, (**b**) LOM of SMSM, (**c**) LOM of OVSM and (**d**) LOM of DMSM. Reproduced with permission [160].

The studies by Conway et al. have resulted in a strong link between the redox potential of the metal/metal oxide pair and the voltage needed for oxygen production on a metal surface. Even for noble metals, the release of oxygen from the metal surface requires the creation of the appropriate metal oxide. The OER mostly occurs on the hydroxide, oxyhydroxide, or oxide layer that spontaneously develops on the electrocatalyst’s surface, according to recent research that has confirmed this conclusion [132,159]. In contrast to AEM, the lattice oxygen mechanism (LOM) releases oxygen by oxidizing the catalyst’s lattice oxygen. In particular, the lattice oxygen itself tends to participate in the OER process to produce oxygen after undergoing oxidation under the OER potential via lattice oxygen redox chemistry. The LOM often outperforms the traditional AEM in terms of explaining the underlying cause of the exceptional catalytic activity seen in specific solid-phase catalysts. As a result, building more effective OER electrocatalysts may be guided by a thorough knowledge of the reaction process and pertinent parameters [160]. The fundamental working of OER is shown in Figure 15.

#### MXene-Based Nanocomposites for OER

Numerous research teams have documented the coupling of sulfides with MXene, which are frequently used as effective catalysts for OER. A FeS_2_@MXene catalyst was made by Xie et al., who also looked at how well it performed for OER. In an alkaline solution, the resulting catalyst exhibited a minor over potential of 240 mV at 10 mA/cm^2^. The scientists discovered that by successfully inhibiting aggregation and modifying the electron density and electrophilicity of the active center, the addition of MXene may significantly enhance the OER activity and stability of FeS_2_ [161].

**Figure 16:**
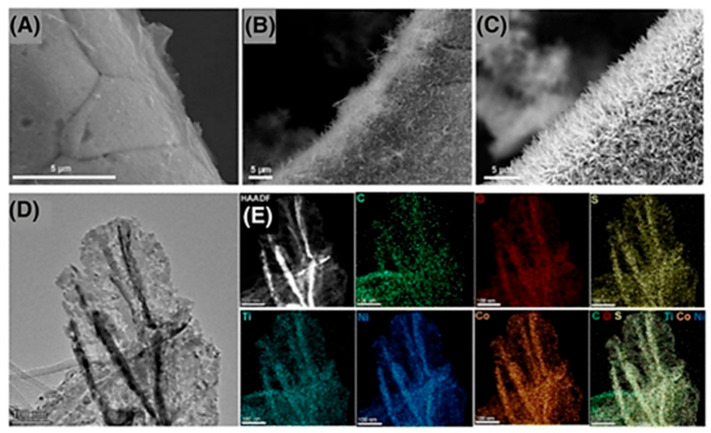

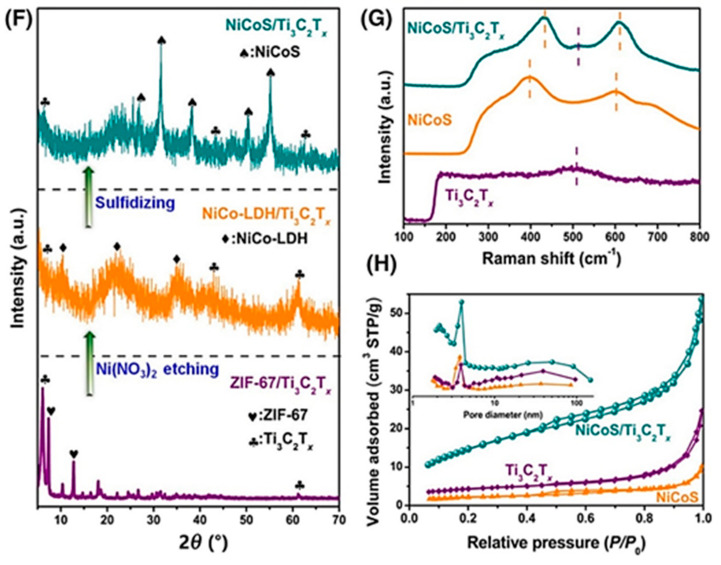
(**A**) NiFeCoP/MXene preparation, (**B**) XRD, (**C**) SEM, (**D**) TEM, and (**E**) EDS mapping, (**F**) LSV curves and (**G**) OER over potential and (**H**) Large surface area. Reproduced with permission [166].

A CoS_2_@MXene catalyst with spatial electron rearrangement, ordered structures, and strong anchoring was described by Han et al. It was discovered that the inclusion of MXene enhanced the intrinsic activity and solidity of CoS_2_ active sites in addition to enriching their density [162]. The design of MXene@Co_3_S_4_/Ni_3_S_2_ on nickel foam, as shown in Figure 16A, and its performance for OER in alkaline (1M KOH) were reported by Li et al. [163]. The synthesis consists of etching and delamination of Ti_3_C_2_T_x_ MXene, heterogeneous development of CoNi-LDH on MXene, represented in Figure 16B, and wet chemical sulfuration Figure 16C–E, to convert it to CoNi sulfide. With an ultralow over potential of 186 mV, long-term stability for 24 h, and exceptional voltammetric cycling stability for 1000 cycles, the hybrid catalyst exhibits exceptional OER performance. According to DFT calculations, the active center with the lowest barrier for *O to *OOH conversion is the Ni atom rather than the Co atom. A porous NiCoS on Ti_3_C_2_T_x_ was reported by Zou et al. as an effective OER catalyst in a related study [164]. Except for using ZIF-67 as the LDH precursor and avoiding nickel foam, the synthesis is identical to that of Li et al., as shown in Figure 16F [163]. NiCoS and Ti_3_C_2_T_x_ MXene were detected by Raman spectroscopy.

The peak shift in Figure 16G of NiCoS demonstrated the significant electrical interaction between NiCoS and Ti_3_C_2_T_x_-MXene. MXene’s two-dimensional structure enables a high surface area, as illustrated in Figure 16H for the hybrid catalyst, making it a viable option for zinc-air battery cathode applications that require long-term durability and minimal charging/discharging over potential. In comparison to the pure phosphide equivalent, the FeP-CoP/Ti_3_C_2_T_x_ composite shows better electrochemical performance for the OER in alkaline media, with a smaller Tafel slope and a lower over potential. Additionally, the combination outperformed several different phosphides made from Prussian blue analogs (PBAs) as well as commercial RuO_2_. More active sites, increased intrinsic activity, and quicker charge transfer kinetics made possible by the addition of Ti_3_C_2_T_x_ MXene were cited as the reasons for the FeP-CoP/Ti_3_C_2_T_x_ composite’s improved performance [165]. The Table 3 represents the OER performance of various MXene based electocatalysts.

**Table 3:** OER performance of MXene-based materials as electrocatalysts.

Electrocatalyst	Tafel slope	Overpotential	References
IrCo@ac-Ti_3_C_2_	60 mV/dec	220 mV	[167]
Ru–FeOOH@ Ti_3_C_2_T_x_	67.7 mV/dec	230 mV	[168]
Cr–FeNi LDH/MXene	54.4	232	[169]
Co–B@Ti_3_C_2_T_x_	53	250	[170]
Co_3_O_4_@Ti_3_C_2_T_x_	118	300	[171]
H_2_PO_2_-/FeNiLDH-V_2_C	46.5	250	[172]
CoFe-LDH/Ti_3_C_2_	50	319	[173]
NiCo-LDH/Ti_3_C_2_T_x_/NF	47.2	223	[174]
CDs@(PdFeNiCo)Nb_x_	62	240	[169]

## 5. Conclusions

Environmental pollution and environmentally friendly power generation are two of the world’s most urgent problems. The MXene-based composites have become a revolutionary class of materials in recent years. They are particularly well-suited for use in the breakdown of antimicrobial pollutants as well as water splitting to obtain hydrogen due to their distinctive mix of excellent electrical conductivity, huge surface area, customizable surface chemistry, and structural plasticity. The review demonstrates how MXenes exhibit synergistic effects that significantly enhance their catalytic and adsorptive properties when incorporated into composite materials with metal oxides, sulfides, polymers, carbon nanostructures, and other 2D materials. MXene-based photocatalysts excel in producing reactive oxygen species, facilitating charge separation, and enabling redox reactions to degrade persistent pharmaceutical residues, such as fluoroquinolones and tetracyclines. These composites are promising candidates for long-term water purification technologies, as they not only demonstrate high degradation efficiency but also excellent recyclability and structural stability after multiple treatment cycles. Their surface terminations and conductive nature reduce over potential, speed up electron transfer, and increase accessibility to the active site. Higher photocurrent densities and steady hydrogen production rates result from the capacity to create heterogeneous structures with semiconductors or metal oxides, which also improve charge carrier mobility and inhibit recombination. Significant safety and environmental risks are associated with the traditional synthesis of MXenes, which involves hazardous chemicals, including hydrofluoric acid. For practical implementation, scalability, repeatability, and surface stability remain major obstacles that must be addressed.

Moreover, the combination of fluorine-free etching and composite design methods holds excellent prospects for overcoming the in-built challenges related to MXene-based materials. These innovations provide the development of environmentally friendly fabrication techniques and the formation of MXene composites with tailored properties, thereby enhancing stability, performance, and functionality. As research in these areas progresses, we can expect more efficient and sustainable MXene materials to emerge, paving the way for broader applications in energy, electronics, environmental, and catalytic industries.

## Data Availability

The data will be available on request.
