# Correction: Irfan et al. Recent Advances in MXene-Based Composites for Their Efficiency in the Degradation of Antibiotics and Water Splitting. *Molecules* 2025, *30*, 3712

**DOI:** 10.3390/molecules31030434

**Published:** 2026-01-27

**Authors:** Syed Irfan, Sadaf Bashir Khan, Sheikha Lardhi, S. AlFaify

**Affiliations:** 1 State Key Laboratory of Environment-Friendly Energy Materials, Southwest University of Science and Technology, Mianyang 621010, China; syedirfan@swust.edu.cn; 2 School of Manufacturing Science and Engineering, Key Laboratory of Testing Technology for Manufacturing Process, Ministry of Education, Southwest University of Science and Technology, Mianyang 621010, China; sadafbashirkhan@swust.edu.cn; 3 Center for Integrative Petroleum Research, King Fahd University of Petroleum and Minerals, Dhahran 31261, Saudi Arabia; 4 Information and Computer Science Department, King Fahd University of Petroleum and Minerals, Al Khobar 31261, Saudi Arabia; 5 Advanced Functional Materials & Optoelectronic Laboratory (AFMOL), Department of Physics, Faculty of Science, King Khalid University, Abha 61421, Saudi Arabia; saalfaify@kku.edu.sa

**References**

In the original publication [1], reference [8] was removed. With this correction, the order of some references has been adjusted accordingly. The authors state that the scientific conclusions are unaffected. This correction was approved by the Academic Editor. The original publication has also been updated.
